# ESIN in femur fractures in children under 3: is it safe?

**DOI:** 10.1007/s00068-022-01965-4

**Published:** 2022-04-08

**Authors:** Raffael Cintean, Alexander Eickhoff, Carlos Pankratz, Beatrice Strauss, Florian Gebhard, Konrad Schütze

**Affiliations:** Department of Trauma-, Hand-, and Reconstructive Surgery, Ulm University, Albert-Einstein-Allee 23, 89081 Ulm, Germany

**Keywords:** Femur fractures, Esin, Pediatric, Toddlers, Treatment

## Abstract

**Background:**

Pediatric femur fractures are a major trauma in children. Different treatment algorithms have been developed but indications for surgical treatment, especially in very young patients, are still controversial. Literature recommends surgical stabilization with elastic-stable intramedullary nailing (ESIN) starting at the age of 3 and non-operative treatment in younger patients. This study sought to present the outcome of patients younger than 3 years of age treated with ESIN for femur fractures.

**Materials and methods:**

Inclusion criteria were patients younger than 3 treated with ESIN in femur fractures. Patient demographics, fracture characteristics, mechanism of injury, outcomes and complications were recorded using charts and X-rays. Primary outcome measures were time to mobility, fracture consolidation and surgical-related complications.

**Results:**

Between 2010 and 2020, 159 patients were treated with ESIN in femur fractures in our institution. A total of 30 patients met the criteria. The mean age was 2.1 ± 0.7 years (13 months–2.9 years). Most common mechanism was fall from standing height (60%). Other mechanisms were motor vehicle accidents as a pedestrian (10%) or as a passenger (10%) as well as direct blow trauma (20%). Femoral shaft fracture was the most common injury (80%). 5 subtrochanteric and one distal metaphyseal femur fractures were found. Mean length of stay was 2.0 ± 1.3 days. Radiographic controls were performed on day 1, 14 and 6 weeks after surgery if not otherwise specified or if complications occurred. 4.6 ± 1.2 (*n* 2–7) X-rays were performed on average after surgery. First radiographic consolidation signs were seen after 2.4 ± 0.6 weeks. Only one child showed surgical-related complication with a leg length discrepancy of 1 cm. In 10% of the patients, shortening after surgery of 1.7 ± 1.4 mm (0.3–3.1 mm) occurred. One child initially treated with traction therapy showed skin irritations and was operated with ESIN. No non-union or ESIN-related complications were found. Mean follow-up was 5.1 ± 4.4 months (4–24 months). First independent mobilization was seen at an average of 3.4 ± 1.1 weeks (2–6 weeks) after surgery. Implant removal was performed after 3.2 ± 1.3 months (2–8 months). No refracture after implant removal occurred.

**Conclusion:**

Early results with ESIN show a reasonable and safe treatment option for femur fractures in toddlers and young children under the age of 3 with easy postoperative care, fast fracture union and early independent mobilization.

## Introduction

Pediatric femur fractures are, with an incidence of 14–20/100.000 per year, a major trauma in children [1–6]. The etiology of the fracture varies with the age of the child. For toddlers and young children, the most common cause of femur shaft fractures is usually a low energy trauma like fall from height and direct blow trauma [3, 6, 7]. In most cases, the trauma is obvious. However, suspicion of child abuse should be kept in mind. Femoral shaft fractures are reported to be rare after intentional trauma with typical injuries being meta- and epiphyseal fractures as well as trauma of the torso and head [8, 9, 30, 31]. Management of femoral shaft fractures in children is affected by a wide number of variables including age and weight of the patient, fracture pattern and associated injuries [1, 4, 6]. Especially in young children under the age of 3, most recommendations suggest non-operative treatment with Pavlik harness or traction followed by spica casting due to high remodeling potential [1, 10]. Starting at the age of three, elastic stable intramedullary nailing (ESIN) is recommended for femoral fractures [4, 7, 11]. As operative and non-operative treatments were described as effective, the impact on family dynamics and burden of care may be relevant in choosing a treatment. Although most studies reported no significant difference in bone healing, several risks and complications and high burden of care on the families in non-operative treatment were found [1, 12, 13]. In the group under 3 years of age, little information is found for the operative treatment with ESIN in femoral shaft fractures. This study was conducted to show the results of ESIN in diaphyseal fractures in children under the age of 3.

## Materials and methods

The study was a retrospective exploratory review at a level-one trauma center. Between January 2010 and December 2019, patients with diaphyseal femoral fractures were identified. We included every patient under 3 years of age treated with open or closed reduction and internal fixation with elastic stable intramedullary nailing. Patients with non-operative treatment as well as patients with no intramedullary nailing were excluded. Distal and proximal femoral fractures treated with Kirschner wires or plating were also excluded. A total of 30 patients, surgically treated using ESIN, were included. All patients were seen weekly for clinical follow-ups after surgery. If not otherwise specified by the surgeon, radiographic controls were performed on day 1, 14 and 42 after surgery as well as before implant removal. Implant removal was planned 3 months after surgery. All complications as well as time to first mobilization and time to fracture consolidation were chart reviewed and categorized.

## Results

### Demographics

We found a total of 159 patients treated with ESIN in femur fractures whereof 30 patients were under the age of 3 and were included in the study. The mean age was 2.1 ± 0.7 years (13 months–2.9 years). We found 20 male and 10 female patients with 14 fractures of the left and 16 fractures of the right femur. Average body mass index (BMI) was 17.1 kg/m^2^ (14.1–20.1 kg/m^2^). One patient initially treated with traction therapy showed skin irritations after 3 days and was operated on the third day. No patient under the age of 3 with non-operative treatment was found in the retrospective search.

### Trauma mechanism

All patients were seen on the day of initial trauma. Seven patients were transferred from other hospitals due to low pediatric trauma experience.

Six patients were admitted in the emergency trauma room due to high energy trauma. Three of these patients were hit by a car as a pedestrian and got an initial CT scan. Three patients were involved in a motor vehicle accident as a passenger. No other major injury was recorded.

Most common mechanism was fall from standing height of around 1.5 m. Ten patients slipped from the parents’ arm. Six patients fell from shopping carts, strollers or other objects with similar heights. Only two patients fell from a changing table.

Six patients suffered direct blow trauma like objects falling, or people accidentally stepping on the extremity. No case of child abuse was found in the emergency department reports as well as follow-up documentation.

### Fracture pattern

The transverse femoral shaft fracture was the most common pattern with 24 patients. In eight of these patients, we found an oblique fracture with mean preoperative femoral shortening of 2.8 cm. No comminuted fracture was found. 5 subtrochanteric fractures were included. Two subtrochanteric fractures were classified as spiral fractures. All of these patients suffered from direct blow trauma (*p* < 0.001). One patient showed a distal diaphyseal fracture without growth plate involvement after falling out of a shopping cart. No significant correlation could be shown between BMI and fracture pattern (*p* = 0.243).

### Surgical technique

Except one patient initially treated with traction therapy, all patients received surgery on the day of admission. Mean time-to-surgery was 4.6 h (2–8 h) after first contact in our emergency room. In all patients we used two Synthes Titanium Elastic Nail System (West Chester, PA) with diameters of 1.5 mm up to 2.5 mm, generally to be up to 2/3 of the diameter of the intramedullary canal. The ESIN are usually pre-bend to secure an intramedullary 3-point-fixation. All patients were operated with retrograde nailing with medial and lateral incisions around 1–2 cm proximal of the growth plate. In three cases, closed reduction failed and a mini-open approach was used to achieve a desirable reduction. During all surgeries, free range of motion (ROM) of the hip and knee was tested after stabilization. After surgery, full weight bearing was allowed. An anterior–posterior as well as lateral X-ray was performed the day after the surgery as it is institutional standard. Most patients were discharged the next day, the mean length of stay was 2.0 days (1–7 days) including the day of admission.

### Follow-up

**Fig. 1:**
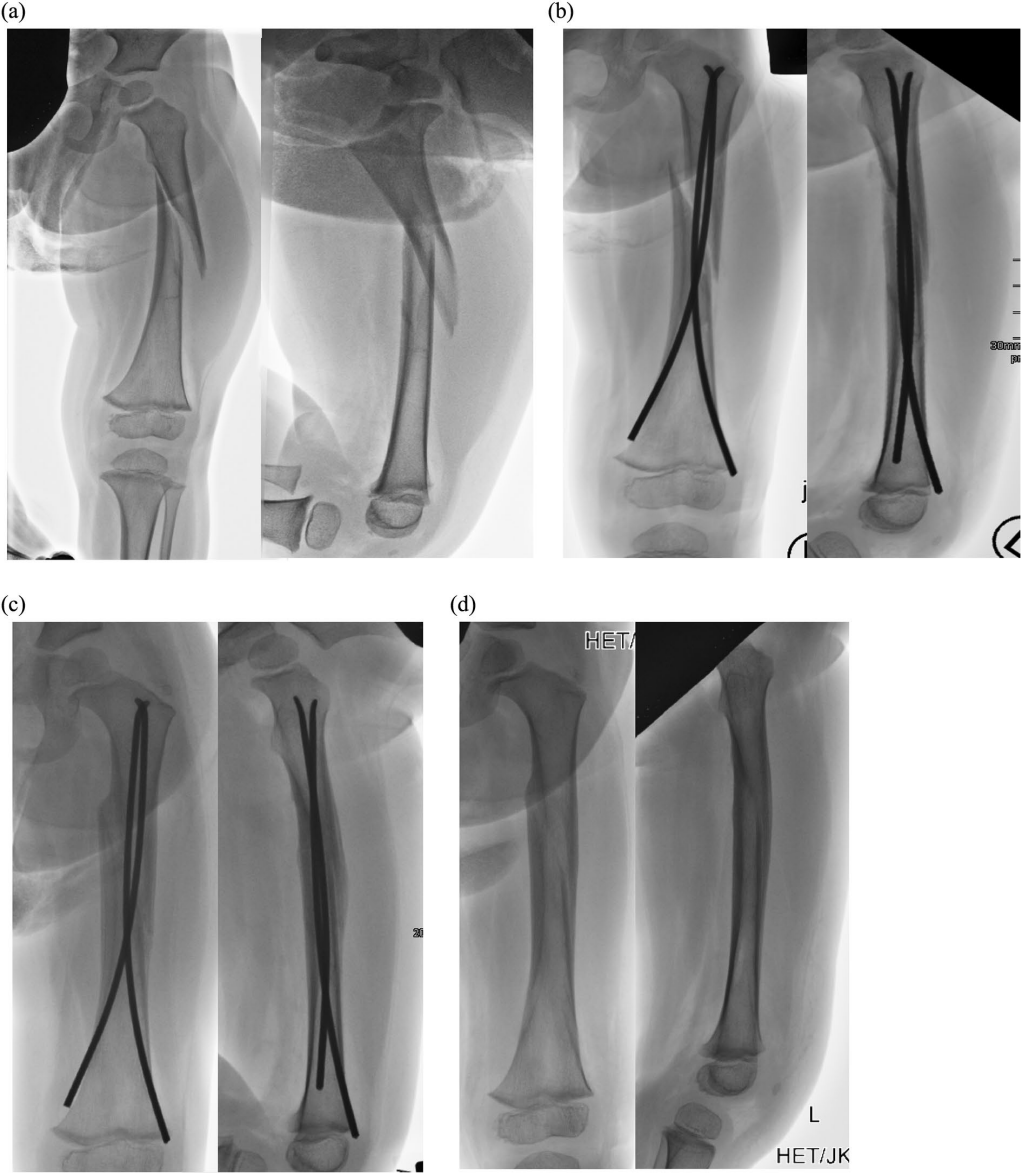
23-Month-old with oblique femur fracture (**a**), 14 days after surgery with first consolidation signs (**b**), after 6 weeks with full consolidation (**c**), after implant removal 8 weeks after trauma (**d**)

The mean follow-up time was 5.1 months (3–24 months) after surgery. No patient was lost during follow-up. All patients were seen weekly after surgery. The average time of follow-up was 5.1 months (3–24 months) with an average of 4.6 (*n* 2–7) radiographic controls in two planes. In 2/3 of the patients, first radiographic consolidation signs were found after 2.4 ± 0.6 weeks. Time to full fracture union was 5.5 ± 1.3 weeks (Fig. 1).

No significant prolongation in bone union could be found. One patient showed a leg-length discrepancy of 1 cm and needed a total clinical follow-up of 24 months with 7 radiographic controls. No further surgical treatment was necessary.

The implant removal was performed 3.2 months (2–8 months) after initial surgery and clinical and radiographic healing was ensured. All implant removals were performed in an outpatient surgery.

### Mobilization

The average time to first independent mobilization was after 3.4 weeks (2–6 weeks). Mobilization was measured based on the ability before trauma after questioning the family. Full independent mobilization was achieved after an average of 5.6 weeks (3–8 weeks). No significant correlation could be shown in BMI and time to first independent mobilization (p = 0.478).

### Complications

No ESIN related complication was found in the charts. One patient initially treated with traction therapy showed severe skin irritation and was operated on the third day after trauma. No non-union was found. After clinical control, we radiographically measured a limb shortening of 1.7 ± 1.4 mm (0.3–3.1 mm) in three cases after surgery without clinical impact.

One patient at the age of 32 months showed a leg-length discrepancy of 1 cm in favor of the broken leg. (Table 1) We performed regular clinical and radiographic follow-ups up to two years after trauma. No further treatment was necessary and no functional impairment was found. No delayed or malunion was found. No refracture after implant removal occurred.

**Table 1:** Child demographics and fracture characteristics in cases with length discrepancies or complications

Child	Age (m)	Trauma	Fracture Type	Shortening (mm)	Overgrowth (cm)	Complication	Union signs (w)	First mobilization (w)	Follow-up (m)	Radiographic controls (n)
1	26	Direct blow	Transverse	0.3	–	–	2	5	4	6
2	27	Fall 1.5 m	Spiral	3.1	–	–	3	4	18	6
3	35	Direct blow	Transverse	1.8	–	–	2	5	5	6
4	32	Direct blow	Spiral	–	1	–	2	6	24	7
5	30	Direct blow	Oblique	–	–	Skin irritation	2	5	4	5

## Discussion

In our study, we found an overall good outcome of pediatric femur fractures after ESIN. Many studies suggest non-operative treatment in pediatric femur fractures under the age of 5 due to quick healing and tremendous potential of remodeling [13–19]. The current German guidelines on pediatric femoral shaft fractures recommend spica casting in patients up to 3 years and ESIN for patients over the age of 3 up to a body weight of 50 kg [4, 11]. The decision to manage these fractures is usually affected by a large number of variables including age, weight, fracture type and associated injuries [4, 7]. Roaten et al. however found considerable variability in treatment and adherence to the guidelines and noted an increased trend toward surgical treatment in patients younger than five years of age [20]. Heffernan et al. included at total of 215 patients in their study comparing the outcome of femoral shaft fractures in children treated with ESIN or spica casting. The ESIN group was significantly older with an average of 4.5 years [13]. Brnjos et al. found in their database research 1181 children treated with ESIN in femoral shaft fractures with an average age of 4.9 years [15]. Lewis et al. reported 32 patients treated with ESIN at 5.3 years of age in average [21]. In the present study, the patients are an average of 2.1 years with no patient being younger than 12 months. Although no study focusing on patients aged 3 and younger surgically treated could be found, some studies compare non-operative and operative treatment with ESIN in pediatric femur fractures.

Considering bone healing, most studies didn’t show any difference between non-operative and operative treatment. Heffernan et al. found similar time to bone healing in a non-operative group (45.1 days) vs. ESIN (44.1 days) without delayed or non-union [13]. Assaghir et al. found a mean time to fracture union of 6.1 weeks [22]. In our study, time to full fracture union was 5.5 weeks after surgery. No non- or malunion was found. Other authors showed similar findings suggesting either treatment method is acceptable [18, 23]. Heffernan et al. however reported earlier independent mobilization in the ESIN group. In their study, the ESIN group showed independent mobilization after 4 weeks compared with 7 weeks in the spica group [13]. Bopst et al. even reported first mobilization after 2.7 days and weight bearing after 14.1 days after surgery [18]. Assaghir found significant differences in favor of ESIN over non-operative treatment in time to full weight bearing (6.2: 7.3 weeks) and rehabilitation time (3.2: 4.0 weeks) [22] Flynn et al. found comparable results [24]. Our study showed similar results. We found first independent mobilization and weight bearing after 3.4 weeks, full independent mobility and weight bearing was achieved after 5.6 weeks.

In the present study, no ESIN related complication could be found. Bopst et al. reported skin irritation or pin exteriorization in 12.3% of the cases [18]. Similar findings were reported by Assaghir with 9.6% of painful nail ends and 1.9% of nail exteriorization [22]. Comparing non-operative and operative treatment, studies show similar complication rates in both methods. Jauquier et al. reported insignificant differences in complication rates between spica and ESIN (10.5%:14.8%) [12]. Assaghir reported, recasting under anesthesia was necessary due to loss of reduction in 13%, 17% redressing for skin irritation and 31% required wedging because of mal-angulation. In the operative group, 4% of the patients with ESIN had painful nail tips and 2% nail exteriorization [22]. Ramo et al. reported revision surgery in 4% of patients in both groups [25]. In a recent study, Brnjos et al. reported 4.4 times more unplanned reoperations in the non-operative group [15]. In our study, we had one case of conversion into surgical treatment due to skin lesions after traction therapy. Flynn et al. showed no significant overgrowth or non-acceptable angulation in the ESIN group, however reported a 1 cm length inequality and 29° varus deformity in the spica group [24]. Jauquier et al. found a total of 26.3% length discrepancy in the non-operative group compared to 18.2% in the ESIN group however mentioning, that both were never clinically significant [12]. Mean shortening was 1.7 mm in average without any clinical significance and in consensus with literature. In the present study, we found one patient with an overgrowth of 1 cm after surgery. In addition to the radiographic consolidation controls, three long leg radiographs were performed to assess further overgrowth. After a clinical and radiographic follow-up of 2 years no further treatment was necessary [13, 18, 19, 22, 25].

In a study of 449 patients, Barnett et al. compared non-operative treatment with spica casting with ESIN. They found higher exposure to radiation as well as anesthesia in the ESIN group [26]. In the contrary, Lewis et al. found no significant differences in number of radiographs between spica (*n* = 3.6) and ESIN (*n* = 3.9, *p* = 0.245) groups [21]. In our study, we performed a comparable amount of radiographs with an average of 4.6 after surgery. Although no non-operative group could be included in the study, we presume a similar number of radiographs would have been necessary. We could not find any significance in fracture pattern or complication rate compared with body weight, which might be a result of the small number of patients we could include. In our patient cohort, no patients body weight was above the 95th percentile. Different studies suggest increased odds of sustaining injury to lower limbs and higher complication rate in overweight children. Weiss et al. demonstrated obesity and high body weight over 50 kg doubles the risk of surgical complications for ESIN in femur fractures [27]. Kessler et al. showed in a cohort study of almost one million patients a higher risk of fractures of the lower limb in obese children, however mentioning no increased risk of femur fractures [28].

Literature suggests both ESIN and non-operative treatment as reliable options for treatment of femur fractures in toddlers and young children. Besides mentioned advantages and disadvantages of both methods, social aspects should be taken in consideration associated with non-operative treatment. Due to the retrospective nature of the study, the direct impact on patients' family life could not be investigated. Gordon et al. reported no significant difference in fracture healing between ESIN and non-operative treatment in femur fractures, however outlines the higher impact on the family in the non-operative group due to higher need of care [1]. Van Cruchten et al. suggested in their systematic review, that overall satisfaction of the parents and the patients after treatment was higher in the ESIN group [32]. Hughes et al. identified impaired mobility due to spica casting of femur fracture as the main factor of family impact. They found in their study, that a mean of 3 weeks off work for caretakers is needed and suggests counseling and planning with the family before cast application if possible [29].

Notable strengths and limitations exist to this study, including its retrospective design. It is the first study providing good clinical results of surgical treatment with ESIN in children under 3. Since we did not have patients with non-operative treatment, no control group could be included. Further, the mean follow-up time does not allow to show long-term complications of leg-length discrepancy after surgery.

## Conclusion

In conclusion, both non-operative treatment and ESIN are reasonable options for treatment of femur fractures in toddlers and young children. Both modalities may lead to acceptable outcomes with each having its specific risks and benefits. With a comparable complication rate and independent mobility but possibly lower impact on family life, ESIN might have a small advantage over non-operative treatment in preschool children. Additional necessity of anesthesia due to implant removal with its risks should be taken in consideration and all treatment options should be discussed with the parents. For patients under the age of one, non-operative treatment is still recommended. Further studies should investigate the impact on family life of spica casting compared to ESIN.
